# Aspirin enhances the therapeutic efficacy of cisplatin in oesophageal squamous cell carcinoma by inhibition of putative cancer stem cells

**DOI:** 10.1038/s41416-021-01499-3

**Published:** 2021-07-27

**Authors:** Zhigeng Zou, Wei Zheng, Hongjun Fan, Guodong Deng, Shih-Hsin Lu, Wei Jiang, Xiying Yu

**Affiliations:** 1 Department of Etiology and Carcinogenesis, National Cancer Center/National Clinical Research Center for Cancer/Cancer Hospital, Chinese Academy of Medical Sciences and Peking Union Medical College, Beijing, China; 2 State Key Laboratory of Molecular Oncology, National Cancer Center/National Clinical Research Center for Cancer/Cancer Hospital, Chinese Academy of Medical Sciences and Peking Union Medical College, Beijing, China; 3 Beijing Key Laboratory for Carcinogenesis and Cancer Prevention, National Cancer Center/National Clinical Research Center for Cancer/Cancer Hospital, Chinese Academy of Medical Sciences and Peking Union Medical College, Beijing, China

**Keywords:** Cancer prevention, Cancer stem cells, Chemotherapy, Oesophageal cancer

## Abstract

**Background:**

Cancer stem cells (CSCs) are related to the patient’s prognosis, recurrence and therapy resistance in oesophageal squamous cell carcinoma (ESCC). Although increasing evidence suggests that aspirin (acetylsalicylic acid, ASA) could lower the incidence and improve the prognosis of ESCC, the mechanism(s) remains to be fully understood.

**Methods:**

We investigated the role of ASA in chemotherapy/chemoprevention in human ESCC cell lines and an N-nitrosomethylbenzylamine-induced rat ESCC carcinogenesis model. The effects of combined treatment with ASA/cisplatin on ESCC cell lines were examined in vitro and in vivo. Sphere-forming cells enriched with putative CSCs (pCSCs) were used to investigate the effect of ASA in CSCs. Assay for Transposase-Accessible Chromatin with high-throughput sequencing (ATAC-seq) was performed to determine the alterations in chromatin accessibility caused by ASA in ESCC cells.

**Results:**

ASA inhibits the CSC properties and enhances cisplatin treatment in human ESCC cells. ATAC-seq indicates that ASA treatment results in remarkable epigenetic alterations on chromatin in ESCC cells, especially their pCSCs, through the modification of histone acetylation levels. The epigenetic changes activate Bim expression and promote cell death in CSCs of ESCC. Furthermore, ASA prevents the carcinogenesis of NMBzA-induced ESCC in the rat model.

**Conclusions:**

ASA could be a potential chemotherapeutic adjuvant and chemopreventive drug for ESCC treatment.

## Background

Oesophageal carcinoma (EC), the third most common cancer and the fourth leading cause of cancer death in China, is estimated to afflict 477,900 new cases while causing 375,000 deaths in a year [1]. Generally, EC consists of two major histopathological types: oesophageal adenocarcinoma and oesophageal squamous cell carcinoma (ESCC). ESCC is the predominant type of EC in the Chinese population, contributing >90% of the cases [2, 3]. To date, the prognosis of ESCC is poor, with an overall survival rate <20% [4].

Although studies of the genomic landscape identified the cancer-driver genes associated with ESCC, there were still a lack of therapeutic targets and precise medical treatments [3, 5–7]. Thus far, conventional surgery, radiation and chemotherapy remain the primary treatment for ESCC but radio- and/or chemoresistance and rapid tumour recurrence caused poor prognosis of ESCC. Hence, findings of new strategies against ESCC such as emphasising prevention and enlarging the clinical application of existing drugs became the focus of clinical and basic research. Cancer stem cells (CSCs), a small sub-group of cancer cells with excessive radio- and chemoresistance, were found in various types of cancers and proved responsible for the recurrence and metastasis of tumours [8]. Previously, we and others identified CSCs in ESCC [9–11]. A large body of evidence suggested that the inhibition of CSCs sensitized tumours to conventional therapies [12–14]. Thus, inhibition of CSCs would improve the outcome of ESCC patients.

Aspirin (acetylsalicylic acid, ASA) is a widely used drug, given its role as an analgesic, antipyretic and agent for cardiovascular prophylaxis. In recent decades, multiple epidemiological studies showed that ASA could help prevent certain cancers, including gastric, pancreatic, breast, ovarian, head and neck, and colorectal cancers [15]. Recently, studies also demonstrated that after diagnosis and chemotherapy, ESCC patients could prolong survival with a regular intake of ASA, indicating that ASA improved the response to conventional treatment [16, 17]. Various mechanisms were identified as contributors to the anti-tumour effects of ASA [18]. As an inhibitor of prostaglandin-endoperoxide synthase 2 (also called cyclooxygenase-2, COX-2), ASA could inhibit the synthesis of prostaglandin E2, which was critical for the maintenance of CSCs through a series of intricate pathways, including NF-κB, PI3K/Akt and Wnt pathways [13, 14]. Furthermore, inhibition of tumour growth and constraint of CSCs by ASA were also reported in a COX-2-independent manner [19, 20]. The acetyl group of ASA could acetylate multiple cellular molecules other than COX-2, such as haemoglobin, histones, transglutaminase and even DNA/RNA, to perform its anti-tumour activities [21, 22]. However, the mechanism(s) by which ASA served as an adjuvant treatment in patients with ESCC and its antitumor effects on ESCC was not fully elucidated. Previously, we and others demonstrated that NMBzA could induce tumorigenesis of rats oesophagi, and it was a valuable animal model for studying ESCC development and progression [23, 24]. By this model, we demonstrated that metformin, a most widely used drug for the management of type 2 diabetes mellitus, had a chemopreventive effect on the carcinogenesis of ESCC [24].

In this study, we investigated the role of ASA as a chemopreventive and adjuvant treatment for human ESCC cells and NMBzA-induced rat ESCC tumours in vitro and in vivo.

## Methods

### Chemicals

NMBzA was synthesized by our laboratory and was found to be >98% pure by high-performance liquid chromatography/mass spectrometry. Dimethyl sulfoxide (DMSO) and ASA were purchased from Sigma-Aldrich Inc. (St. Louis, MO, USA).

### Animals

All animal experiments and protocols were approved by the Ethical Committee of the National Cancer Center/Cancer Hospital, Chinese Academy of Medical Sciences. Female BALB/c nude mice and male Fisher 344 (F344) rats (3–4 weeks old) were purchased from Vital River Laboratory Animal Technology (Beijing, China). Mice and rats were housed five and two per cage under standard conditions (24 ± 2 °C, 20 relative humidity, 12-h light/dark cycles) and given access to standard rodent maintenance feed (Keao Xieli Feed, Beijing, China) and water ad libitum. Hygienic conditions were maintained by weekly cage changes. After completion of experiments, we sacrificed mice and rats by inhalation of anaesthetics with CO_2_.

### Cell culture and ESCC xenograft in nude mice

ESCC cell lines (KYSE150, KYSE180, KYSE410 and KYSE510) were gifts from Dr. Y Shimada of Kyoto University and cultured in RPMI-1640 medium with 10% foetal bovine serum. The above cell lines were authenticated under short tandem repeat analysis by Beijing Microread Genetics and matched in DSMZ data back in 2016. All the cell lines were stored in liquid nitrogen and passaged for <6 months in our laboratory. These cell lines were cultured at 37 °C with 5% CO_2_.

The freshly prepared ESCC cell lines (1 × 10^6^ cells/each) were injected subcutaneously into the right armpit of female nude mice (4 weeks old). The mice were monitored every other day for palpable tumour formation. The tumour volume was estimated by calibrating the length (*L*) and the width (*W*) of the palpable tumour (volume ≈ 0.5 × *L* × *W*^2^). At week 6, 24 mice were randomly divided into four separate groups as follows (six mice per group): negative control (NC), 2 g/L ASA in water (ASA), 5 mg/kg cisplatin once per week (Cis-diamminedichloroplatinum, DDP) and 5 mg/kg cisplatin once per week plus 2 g/L ASA in water (ASA+DDP). The doses of ASA and DDP used in this study were within the safe range. To determine tumour formation, the mice were killed on the 61st day after transplantation.

### Analyses of cell viability, proliferation, mitoxantrone efflux, apoptosis and colony formation

Cell viability was determined with Cell Counting Kit-8 (Dojindo, Kumamoto, Japan). The number of viable cells per well was measured by the absorbance (450 nm) using the Multiskan GO Spectrophotometer (Thermo Fisher Scientific, Karlsruhe). The proliferation of ESCC cells was monitored using the xCELLigence Real-Time Cell Analyzer (RTCA)-MP system (Acea Biosciences/Roche Applied Science) as described [3]. A total of 2 × 10^3^ cells in 100 μL of culture medium were seeded in E-Plate 96 (Roche Applied Science), which was then locked in the RTCA-MP device at 37 °C with 5% CO_2_. The medium was exchanged with a fresh medium containing DDP and/or different concentrations of ASA 24 h later. Cell apoptosis was analysed using Annexin V Assay Kit (Invitrogen) and the terminal deoxynucleotidyl transferase dUTP nick-end labelling (TUNEL) Staining Assay Kit (Beyotime, China) according to the manufacturer’s instruction.

The efflux assays were performed as previously described [9]. KYSE180, KYSE410 and KYSE510 cells were treated with different concentrations of ASA (0, 2.5 or 5 mM) for 24 h after being seeded in six-well plates. Then, they were treated with 200 nM mitoxantrone for 1 h. The mitoxantrone fluorescence was detected using a 670 nm bandpass filter by flow cytometry.

For plate colony formation assay, cells were plated at a density of 500 cells per well in six-well plates in triplicate. After being cultured for 14 days, the plates were stained with 0.5% crystal violet and colonies were examined and automatically calculated by G:box (Syngene).

### ALDEFLUOR assay

Aldehyde dehydrogenase 1 (ALDH1) activity was analysed using ALDEFLUOR^TM^ Kit (Stem Cell Technology, Canada) following the manufacturer’s protocol. In brief, 1 × 10^6^ cells in 1 mL of ALDEFLUOR buffer was mixed with BAAA at a concentration of 1.5 mM for 45 min at 37 °C. Each sample of cells was treated under identical conditions with 15 μM of the ALDH inhibitor Diethylaminobenzaldehyde (DEAB) to serve as an NC. Analysis of the samples was determined by flow cytometry using FACScan instrument equipped with a 488/513 channel to collect the signal of the fluorescent dye (BAAA and BAA).

### Tumour sphere culture and sphere-forming assay

For tumour sphere culture, ESCC cells were seeded as a single cell at a density of 4 × 10^3^ cells per well in ultra-low attachment six-well plates (Corning, NY, USA) under serum-free DMEM/F12 (Gibco) medium supplemented with 4 μg/mL heparin, 20 ng/mL basic fibroblast growth factor, 20 ng/mL epidermal growth factor (PeproTech Inc., Rocky Hill, USA), 5 μg/mL insulin (Sigma-Aldrich) and 1× B-27 (Invitrogen). After incubation for 10 days, small clusters of ESCC cells could be observed under a microscope and harvested with a nylon mesh (BD Falcon). For the sphere-forming assay, the ESCC cells were plated at a density of 2 × 10^3^ cells per well in 6-well plates with triplicate. The number of tumour spheres in each well was counted carefully under the microscope.

### Immunoblotting, quantitative real-time PCR (q-PCR) and RNA interference (RNAi)

Immunoblotting, immunofluorescence and q-PCR were performed as previously described [25]. In brief, for immunoblotting, samples were lysed in RIPA buffer (Beyotime) supplemented with a complete protease inhibitor cocktail (Thermo Scientific) and a total of 30 μg of protein was resolved on precast sodium dodecyl sulfate-polyacrylamide gel electrophoresis gels for electrophoresis and immunoblotting. The primary antibodies were purchased as follows: anti-Sox2 (Abcam, ab92494), anti-Nanog (Cell Signaling Technology, 4903), anti-Oct4 (Cell Signaling Technology, 2750), anti-Akt (Santa Cruz Biotechnology, sc-5298), anti-p-Akt (Santa Cruz Biotechnology, sc-377556), anti-pATM (Abcam, ab81292), anti-cleaved caspase-3 (Cell Signaling Technology, 9664), anti-acetyl-H3K9 (Cell Signaling Technology, 9649), anti-acetyl-H3K27 (Cell Signaling Technology, 8173), anti-Bim (Cell Signaling Technology, 2933) and anti-β-actin (Sigma-Aldrich, A5441). The secondary antibodies were purchased as follows: goat anti-rabbit IgG antibody (Beyotime) and goat anti-mouse IgG antibody (Beyotime). The protein bands were photographed using ImageQuant™ LAS 4000.

For q-PCR, total RNA was extracted using the Trizol reagent (Ambion, USA) and reverse-transcribed to complementary DNA using the PrimeScript™ RT Reagent Kit (Takara, Dalian, China). Q-PCR was carried out using the SYRB Premix Ex Taq™ Perfect Real-Time system (Takara). The primers used are listed in Supplementary Table S1. The expression levels were normalized to that of the housekeeping gene *GADPH*.

For RNAi, cells were cultured in six-well plates with 1 × 10^6^ cells per well. The cells were transfected with small interfering RNA (siRNA) against Bim using Lipofectamine 3000 Kit (Invitrogen) according to the manufacturer’s instructions. The siRNA target sequences were as follows:

siBim-1: 5′-CAUGAGUUGUGACAAAUCAACACAA-3′ and 5′-UUGUGUUGAUUUGUCACAACUCAUG-3′.

siBim-2: 5′-UGAGUGUGACCGAGAAGGUAGACAA-3′ and 5′-UUGUCUACCUUCUCGGUCACACUCA-3′.

### ATAC-seq

Assay for Transposase-Accessible Chromatin with high-throughput sequencing (ATAC-seq) samples was prepared as previously [26] using ~10,000 living cells. The cells were lysed in 1× Lysis Buffer and TruePrep™ DNA Library Prep Kit V2 for Illumina (Vazyme Biotech) was used to construct the transposase-treated libraries. The mass concentration and molar concentration of libraries were detected by Qubit 3.0 Fluorometer and StepOnePlus™ Real-Time PCR system, respectively, and lengths of inserted fragments were detected with Agilent HS 2100 Bioanalyzer. Qualified libraries were sequenced using Illumina HiSeq X ten platform in pair-end 150 bp style.

Raw sequencing data were filtered for Adaptor-polluted or low-quality reads to get the clean data. Clean data were aligned on GRCh38 using Bowtie2, and visualized by Integrative Genomics Viewer. Peaks corresponding to the open region in the genome were called using MACS2. The enrichment analysis of GO term (http://geneontology.org/) or KEGG pathway (http://www.kegg.jp/) was based on a hypergeometric test with the threshold *q* < 0.05, to find the significant enrichment of detected genes.

### Establishment of oesophageal cancer model in rat using NMBzA and ASA administration

NMBzA (20 mg) was dissolved in 1 mL DMSO and then was diluted with phosphate-buffered saline (PBS) into the concentration of 1.2 mg/mL for injection. ASA was dissolved in water at the concentration of 2 g/L. Seventy-eight male F344 rats were given subcutaneous injections with 0.25 mL/kg of 6 % DMSO in PBS or 0.3 mg/kg NMBzA three times per week. They were randomly divided into three separate groups as follows: 0.25 mL/kg of 6% DMSO in PBS (DMSO group, *n* = 14), 0.3 mg/kg NMBzA (NMBzA-treated group, *n* = 32), 0.3 mg/kg NMBzA plus 2 g/L ASA in water (NMBzA plus ASA treated group, *n* = 32). Six rats of the DMSO group and eight rats from other groups were sacrificed at week 25, whereas at week 35, eight rats of the DMSO group and 24 rats from other groups were sacrificed. Each oesophagus was opened longitudinally, and the tumours were counted, mapped and sized. Tumours >0.5 mm in diameter were counted. The volume of the tumours was calculated using the standard formula: the length × width × height × *π*/6. Each oesophagus was cut into three parts (upper, middle and lower) and embedded in paraffin.

### Histological and immunohistochemical analyses

The haematoxylin- and eosin-stained slide was made from the three parts of each oesophagus. Each viewing field was categorised into one of five histologic categories: normal epithelium, hyperplasia, dysplasia, papilloma and carcinoma. Diagnostic criteria were the same as those of Pozharisski [27] and the International Harmonization of Nomenclature and Diagnostic Criteria [28].

Immunohistochemistry (IHC) was performed on formalin-fixed, paraffin-embedded tissue as previously described [25]. The following primary antibodies were employed overnight at 4 °C: anti-Ki67 (Abcam, ab16667, 1:200), anti-Sox2 (Abcam, ab92494, 1:200), anti-cyclin D1 (Abcam, ab16663, 1:200), anti-PCNA (Cell Signaling Technology, 13110, 1:3000), anti-Bim (Cell Signaling Technology, 2933, 1:200) and anti-COX-2 (Cell Signaling Technology, 12282, 1:500). Immunostaining was performed using the PV-9001 or PV-9002 Polymer Detection System with diaminobenzidine according to the manufacturer’s recommendations (GBI, USA) and subsequently counterstained with hematoxylin. Slides without the addition of the primary antibody served as an NC. Six non-contiguous randomly selected fields in each slide were photographed under ×100 and ×400 magnification. Cytoplasm or nuclear stain-positive cells were analysed by Image-pro plus 6.0.

### Statistical analysis

Statistical analysis of differences between samples was performed using Student’s *t* tests and results were considered to be significant at *P* < 0.05. The analysis was conducted using GraphPad Prism 7.0. The results were presented as mean ± SD unless otherwise stated.

## Results

### ASA promoted the therapeutic effect of DDP in human ESCC cells

**Fig. 1:**
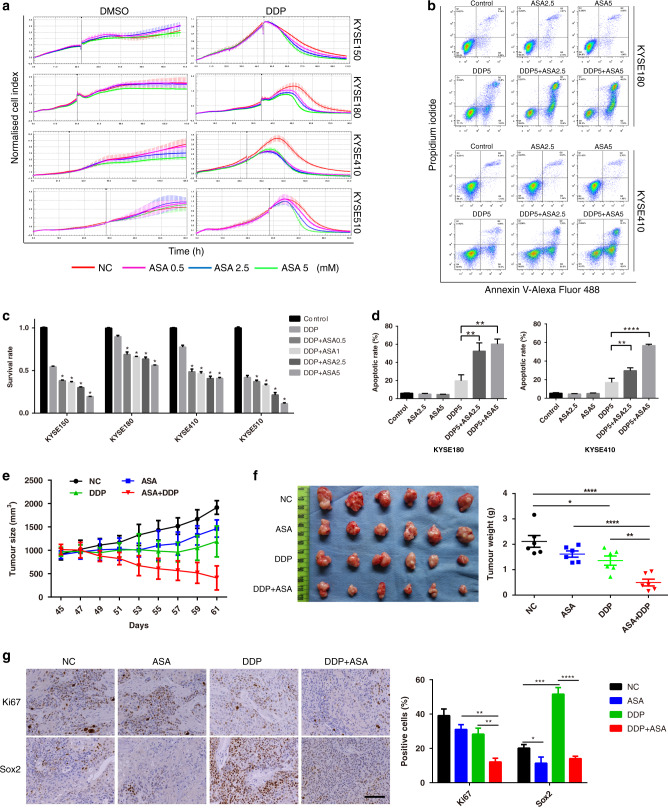
ASA improves the effect of DDP in the treatment of ESCC. **a** RTCA analysis of ESCC cells exposed to indicated concentrations of ASA with DMSO (left) or 5 μg/mL DDP (right). **b** The survival rate of ESCC cells treated with 5 μg/mL DDP or combined with ASA for 24 h (*n* = 3). All *p* values are compared with DDP treatment alone. **c**, **d** Representative of apoptosis analyses of KYSE180 and KYSE410 cells exposed to DDP, ASA or the combination. **d** Percentage of apoptotic cells in the indicated groups (*n* = 3). **e** Tumour growth of KYSE410 xenografts subjected to the indicated treatments: vehicle, ASA (2 g/L in drink water), DDP (5 mg/kg) or the combination (*n* = 6). **f** Images and tumour weights of KYSE410 xenografts (*n* = 6). **g** Representative images (left) and percentage of positive cells (right) of IHC staining against Ki67 and Sox2 on xenograft tumour sections (*n* = 6). Scale bars, 100 μm. In all the panels, error bars indicate means ± SD; *p < 0.05, **p < 0.01, ***p < 0.001 and ****p < 0.0001. Student’s *t* test.

DDP is the most widely employed drug in the treatment of ESCC. To investigate the chemotherapeutic adjuvant potential of ASA in ESCC, four ESCC cell lines (KYSE150, KYSE180, KYSE410 and KYSE510) were treated with ASA and/or DDP. The therapeutic responses were visualized using RTCA. As shown in Fig. 1a, the growth of all ESCC cells was not significantly influenced by ASA (left). DDP alone decreased the number of viable ESCC cells, but combined with ASA, it could trigger a more robust loss of ESCC cells in a dose-dependent manner (right). Consistently, cell viability assay showed that when compared to DDP treatment, a combination of DDP and ASA significantly decreased ESCC cell viability in a dose-dependent manner (Fig. 1b). Since KYSE180 and KYSE410 cells were more resistant to DDP than KYSE150 and KYSE510 cells (Fig. 1b), we carried out further experiments on these two cell lines. Fluorescent-activated cell sorting (FACS) analysis showed that combined DDP/ASA treatment in ESCC cells significantly increased cell death within 24 h as compared with DDP treatment alone (Fig. 1c, d).

The synergistic therapeutic effects of combined DDP/ASA treatment in ESCC cells in vitro prompted us to test the efficacy of DDP/ASA against KYSE410 xenografts in vivo. Drug treatments were initiated when tumours reached an average volume of ~1000 mm^3^ (~45 days after subcutaneous inoculation). As shown in Fig. 1e, f, ASA treatment did not effectively suppress xenograft growth. DDP treatment alone slowed the tumour growth but did not cause significant tumour shrinkage compared with the beginning of treatment. However, the combination led to intense tumour regression. The expression of Ki67 tested by IHC also revealed that the combination treatment significantly arrested tumour growth (Fig. 1g). Similar results were also obtained in KYSE150 xenograft tumour experiments (Supplementary Fig. S1). Taken together, these results indicated that ASA could potently enhance the sensitivity of ESCC cells to DDP in vitro and in vivo.

### ASA suppressed the CSC properties of human ESCC cells

**Fig. 2:**
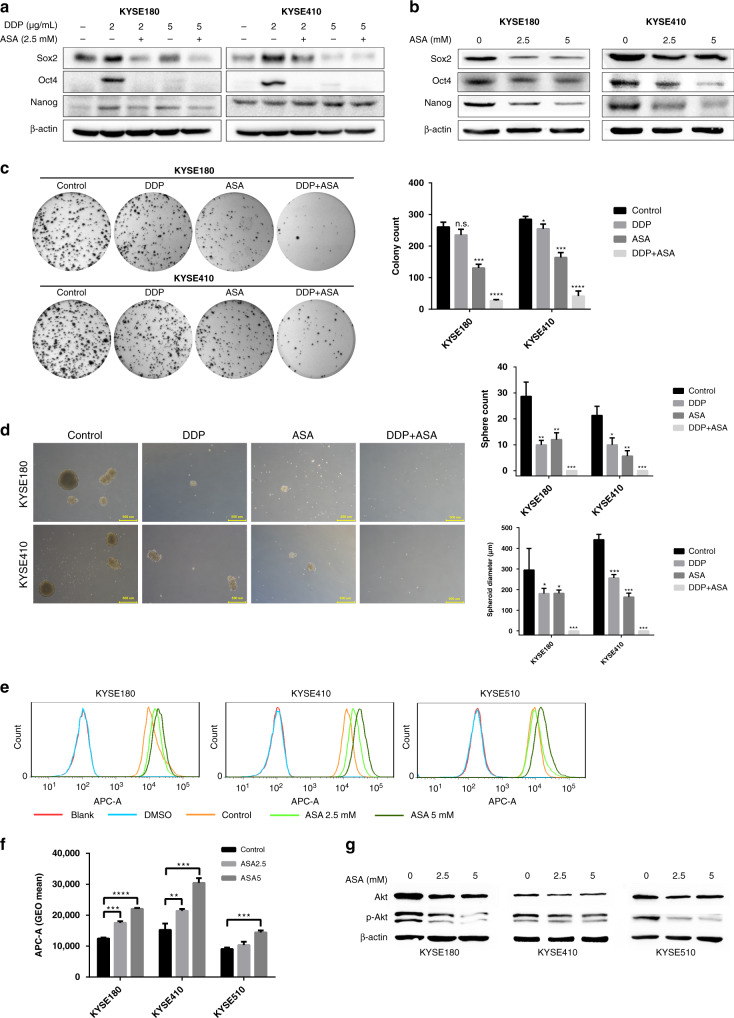
ASA inhibits the stemness of ESCC cells. **a** Immunoblot analysis for stem cell markers in KYSE180 and KYSE410 cells treated with the indicated drug combinations for 72 h. **b** Immunoblot analysis for stem cell markers in KYSE180 and KYSE410 cells treated with ASA at the indicated concentrations for 48 h. **c** Representative images from colony formation assay and the colony count of KYSE180 and KYSE410 cells pretreated with 2 μg/mL DDP, 5 mM ASA or the combination for 48 h (*n* = 3). **d** Representative images from spheroid formation assay and the spheroid count/diameters of KYSE180 and KYSE410 cells pretreated with 2 μg/mL DDP, 5 mM ASA or the combination for 48 h (*n* = 3). **e** Representative flow cytometry analysis of mitoxantrone efflux in untreated and ASA-treated cells. Mitoxantrone was examined at 670 nm, with blank and DMSO as a negative control. **f** Statistic analysis of Geo Mean of the fluorescence collected from three parallel experiments. **g** Inhibition of phosphorylation of Akt determined by immunoblot analysis. In all the panels, error bars indicate means ± SD; *p < 0.05, **p < 0.01, ***p < 0.001, ****p < 0.0001; n.s. not significant. Student’s *t* test.

CSCs are reported to be responsible for tumour chemoresistance [29]. Given the fact that ASA could increase the cytotoxicity of DDP against human ESCC cells in vitro and in vivo, we next investigated whether ASA could affect the CSCs of ESCC cells. Consistent with previous reports [30, 31], western blot analysis demonstrated that low-dose DDP treatment (2 μg/mL for 72 h) could enhance the expression of stemness-associated genes in KYSE180 (*Sox-2*, *Oct4* and *Nanog*) and KYSE410 cells (*Sox-2* and *Oct4*, Fig. 2a), suggesting that CSCs could be enriched by DDP treatment. Similarly, IHC analysis of KYSE410 xenografts revealed a dramatic enrichment of Sox2-positive cells in the DDP treatment group (Fig. 1g). However, this enrichment effect was blocked by ASA treatment (Figs. 1g and 2a), suggesting that ASA could reduce CSC properties in ESCC. Actually, ASA alone is sufficient to reduce the expression of stemness-associated genes (Fig. 2b) and the proportion of Sox2-positive cells in xenografts (Fig. 1g).

Previously, we and others demonstrated that CSCs displayed a stronger capability of colony-formation, spheroid-formation and cell efflux activity than non-CSCs [32–36]. Therefore, we next investigated whether ASA could affect these stemness-associated functional phenotypes in KYSE180 and KYSE410 cells. Both cells were pretreated with 2 μg/mL DDP, 5 mM ASA or combined treatment for 48 h, and then colony- and spheroid-formation assays were performed. Compared with untreated control cells and DDP-treated cells, pretreatment with ASA in KYSE180 and KYSE410 cells resulted in a significant reduction of colony formation and combination treatment showed the strongest inhibitory effects (Fig. 2c). Spheroid-formation assays also found that cells pretreated with DDP and ASA formed smaller and fewer spheroids, but the combination could prevent any measurable spheroid (Fig. 2d). In addition, we performed a mitoxantrone efflux assay to examine the effect of ASA on the efflux activity of ESCC cells. As shown in Fig. 2e, f, ASA-treated ESCC cells displayed greater emission fluorescence at 670 nm than ASA-untreated cells, indicating that ASA affected the cell efflux activities in ESCC cells. As we and others previously showed that PI3K/Akt played critical roles in regulating the cell efflux activities of CSCs in ESCC cells [9, 37], we also examined whether ASA was involved in affecting the PI3K/Akt signal pathway. Immunoblotting analysis indicated that treatment with 2.5 or 5 mM of ASA in KYSE180, KYSE410 and KYSE510 cells for 48 h resulted in the dramatic suppression of phosphorylation level of Akt (p-Akt) when compared with controls (Fig. 2g).

Collectively, these results indicated that treatment of ASA in ESCC cells reduced their CSC properties, resulting in ASA enhancing the efficacy of chemotherapeutic drugs such as DDP in the treatment of ESCC cells.

### ASA increased the death of pCSCs in ESCC cells

**Fig. 3:**
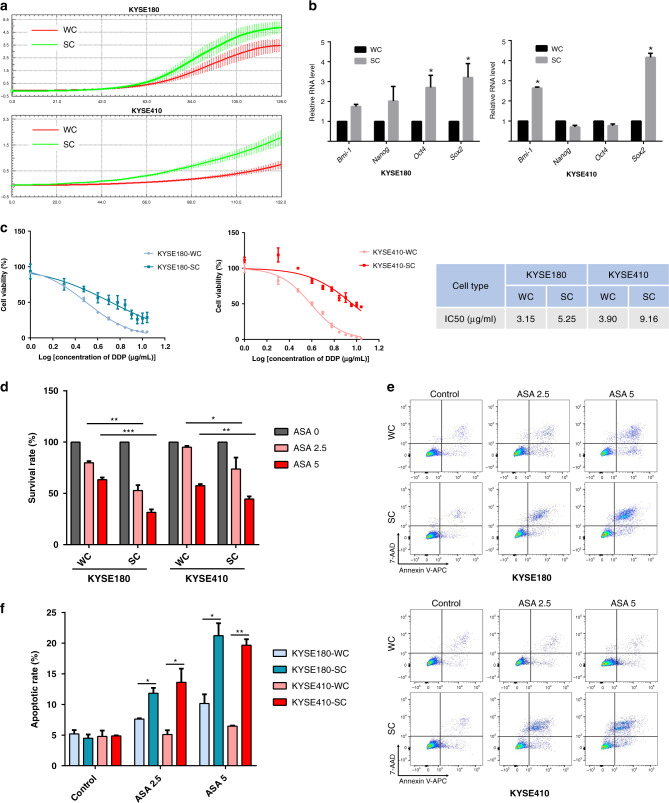
ASA promotes cell death in pCSCs of ESCC. **a** RTCA analysis showing stronger proliferation activity in SCs. **b** qRT-PCR results showing the expression of stem cell markers in WCs and SCs (*n* = 3, normalized to GAPDH expression). **c** Growth-inhibitory curves of WCs and SCs of KYSE180 and KYSE410 cells treated with DDP (*n* = 5) and the IC50 values are measured. **d** The survival rate of WCs and SCs of ESCC cells treated with ASA at the indicated concentrations for 48 h (*n* = 3). **e**, **f** Representative images of Annexin V assay (**e**) and quantification (**f**) show the percentage of apoptotic cells of WCs and SCs following treatment with ASA for 48 h (*n* = 3). In all the panels, error bars indicate means ± SD; *p < 0.05, **p < 0.01, and ***p < 0.001. Student’s *t* test.

In view of the suppression of ASA in CSC properties of ESCC cells, we next studied how ASA was directed against CSCs. Firstly, as tumour spheres are thought to be spherical formations developed from the proliferation of one cancer stem/progenitor cell [38], we performed spheroid-formation assays to enrich and isolate pCSCs from ESCC cells. The tumour spheres formed from KYSE180 and KYSE410 cells were collected and digested into single-cell suspension, termed as sphere-forming cells (SCs). RTCA analyses showed that compared with the whole-cell population (termed as WCs), SCs exhibited more potent proliferation ability (Fig. 3a). We further detected the expression of stemness-associated genes and the results showed that KYSE180-SC expressed higher levels of messenger RNAs of *Bmi-1*, *Nanog*, *Oct4* and *Sox-2*, while KYSE410-SC expressed higher levels of *Bmi-1* and *Sox-2* (Fig. 3b). Indeed, SCs displayed stronger resistance to DDP, with a higher IC50 (half-maximal inhibitory concentration) than WCs (Fig. 3c). These results indicated that SCs of KYSE180 and KYSE410 cells represented CSC properties.

Subsequently, we compared the inhibitory effects of ASA in KYSE180 and KYSE410 cells (KYSE180-WCs and KYSE410-WCs) and their corresponding SCs (KYSE180-SCs and KYSE410-SCs). As shown in Fig. 3d, inhibition of viabilities of KYSE180-SCs and KYSE410-SCs by ASA treatment was more remarkable than that of KYSE180-WCs and KYSE410-WCs and this effect was shown to be dose-dependent. Furthermore, FACS analysis indicated that cell death was notably higher in the SC population compared with the WC population following incubation with ASA (Fig. 3e, f). TUNEL assay also showed that ASA treatment resulted in significant cell death in SCs (Supplementary Fig. S2A). Consistently, the ALDEFLUOR assay, which identified and enumerated potential CSCs expressing high levels of ALDH in KYSE180-WCs, revealed that the percentage of cells with high ALDH activity was significantly reduced by ASA treatment when compared with controls (Supplementary Fig. S2B). These results indicated that ASA could inhibit both SCs and non-SCs, but SCs are more susceptible to ASA in promoting cell death. Importantly, ASA combined with DDP could further enhance the inhibitory effect on SCs (Supplementary Fig. S2C).

Previous studies demonstrated that the main action of ASA in cancer cells that caused cell death was its irreversible inactivation of COX-2 [14, 39]. Hence, we examined whether ASA induced cell death in ESCC cells via a COX-2-dependent pathway by comparing actions of ASA and NS-398, a selective inhibitor of COX-2, in KYSE180 and KYSE410 cells. As shown in Supplementary Fig. S3A, B, unlike the treatment of ASA, treatment of NS-398 did not promote cell death and inhibit sphere formation in ESCC cells significantly, suggesting that the effects of ASA might be attributed to COX2-independent pathways.

### ASA altered the chromatin structure and accessibility of pCSCs in ESCC cells

**Fig. 4:**
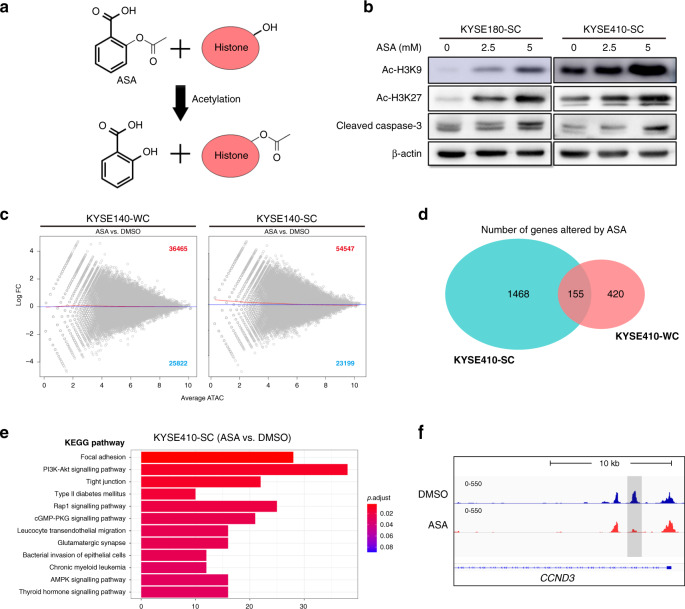
ASA alters the chromatin accessibility of pCSCs in ESCC cells. **a** Schematic diagram of acetylation of serine hydroxyl groups on histone by ASA. **b** Immunoblotting for cleaved caspase-3, Ac-H3K9 and Ac-H3K27 in SCs in the presence of ASA. **c** MA plots showing log 2 fold change of chromosome-accessibility peaks of ATAC-seq analysis in KYSE410 between ASA or DMSO treatment. **d** Venn diagram for genes with altered chromatin accessibility upon ASA-treatment in KYSE410-SCs and WCs. **e** KEGG pathway analysis among genes altered by ASA for chromatin accessibility in KYSE410-SCs. **f** Genome browser image showing ATAC-seq analysis of *CCND3* with a grey box indicating changed chromatin accessibility upon ASA treatment.

To determine the potential mechanism(s) by which ASA inhibited pCSCs in ESCC cells, we then focused on the acetyl group of ASA that was shown to acetylate several proteins including histones through a transacetylation (Fig. 4a) [40, 41]. Immunoblotting analysis revealed that histone H3 was significantly acetylated and cleaved caspase-3 was significantly increased in ESCC cells following ASA treatment (Fig. 4b). Previous studies showed that ASA specifically inhibited the acetyltransferase activity of EP300 by competition with acetyl CoA and blocked the acetylation of histones [42, 43], indicating that alterations of histone modifications by ASA in ESCC cells were complicated and profound. To determine the alterations in chromatin structure and accessibility across the genome caused by ASA in ESCC cells in detail, ATAC-seq was performed. After 24 h of DMSO (control) or ASA treatment, KYSE410-WCs and KYSE410-SCs were generated to two replicates and performed for ATAC-seq.

The ATAC-seq experiments identified a number of peaks that were markedly altered in each replicate of cells, especially in KYSE410-SCs treated with ASA (Supplementary Fig. S4A). Bioinformatic analysis indicated that distributions of the peaks were enriched at intergenic, intron and promoter regions in all conditions tested (Supplementary Fig. S4B). As the different peaks identified were further analysed by MAnorm, broad alterations in chromatin structure and accessibility were observed in KYSE410-WCs and KYSE410-SCs treated with ASA. In KYSE410-SCs, 54,547 regions were found to become more accessible and 23,199 regions were found to become less accessible following treatment with ASA (Fig. 4c and Supplementary Table S2). In contrast, 36,465 regions gained and 25,822 regions lost chromatin accessibility in KYSE410-WCs treated with ASA (Fig. 4c and Supplementary Table S2). Although ASA treatment resulted in remarkable changes of chromatin structure and accessibility in both cell populations, the effects on KYSE410-SCs were more significant, where more of the chromatin regions became altered (Fig. 4c). We further analysed genes with changed chromatin structure and accessibility upon ASA treatment and found that 1623 or 575 differential genes were altered in KYSE410-SCs or KYSE410-WCs, respectively (Fig. 4d and Supplementary Table S3). Among these genes identified, only 155 genes were shared in KYSE410-SCs or KYSE410-WCs (Fig. 4d).

Notably, the KEGG pathway analysis showed that the genes altered in KYSE410-WCs could not be enriched well (*P* > 0.05) (Supplementary Fig. S4C). In contrast, the analysis of genes altered in KYSE410-SCs revealed high enrichment in growth and migration-related signals such as PI3K-Akt signal pathway (Fig. 4e), consistent with the results obtained in Fig. 2g. As a downstream of Akt, *CCND3* became less accessible due to ASA-induced chromatin changes (Fig. 4f). Ontology analysis (GO analysis) of genes with altered structure and accessibility in ASA-treated KYSE410-SCs showed that cell metabolic and biological regulation were enriched (Supplementary Fig. S4D). Taken together, these results indicated that ASA functioned through its transacetylation on chromatin directly or indirectly by modifying acetylation of nucleosomes/histones and/or DNA that altered the chromatin structure and accessibility, resulting in extensive changes of the epigenetic/biological processes in ESCC cells, especially pCSCs.

### ASA caused pCSCs death by the upregulation of BIM expression

**Fig. 5:**
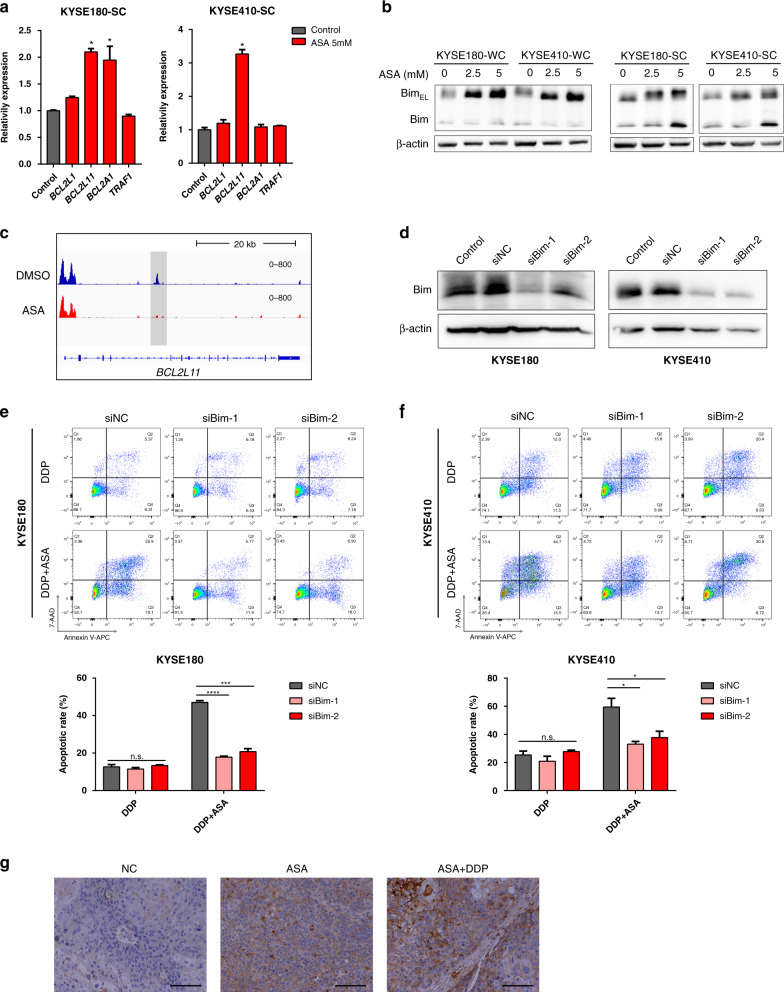
ASA induces cell death in pCSCs of ESCC dependent on Bim up-regulation. **a** qRT-PCR results showing the expression of indicated genes in SCs upon treatment with 5 mM ASA (*n* = 3, normalized to GAPDH expression). **b** Immunoblotting for Bim in WCs and SCs in the presence of ASA. **c** Genome browser image showing ATAC-seq analysis of *BCL2L11* with a grey box indicating changed chromatin accessibility upon ASA-treatment. **d** Western blot analysis confirming siRNA-mediated Bim knockdown in KYSE180 and KYSE410 cells. **e, f** Representative images of Annexin V assay and quantification show the percentage of apoptotic cells of KYSE180 (**e**) and KYSE410 (**f**) cells following 24 h treatment with DDP (5 μg/mL) or combined with ASA (5 mM) after knocking down Bim expression (*n* = 3). **g** Representative images of IHC staining against Bim on xenograft tumour sections following treatment with ASA or combined with DDP. Scale bars, 100 μm. In all the panels, error bars indicate means ± SD; *p < 0.05, ***p < 0.001 and  ****p < 0.0001; n.s. not significant. Student’s *t* test.

To ascertain how ASA treatment resulted in cell death in ESCC cells, we further interrogated the ATAC-seq data. Although no clear cell death-related pathways were enriched using KEGG analysis, ATAC-seq revealed several cell death-related genes with altered chromatin structure and accessibility upon ASA treatment in ESCC cells, especially SCs. These genes were *BCL2L1*, *BCL2L11*, *BCL2A1* and *TRAF1*. We performed qRT-PCR and verified the expression of these genes in KYSE180-SCs and KYSE410-SCs. Among these genes, *BCL2L11*, also called BIM, which encodes a proapoptotic protein belonging to the Bcl-2 family, was significantly increased in both KYSE180-SCs and KYSE410-SCs after treatment with ASA (Fig. 5a). Immunoblotting analysis indicated that ASA treatment resulted in increased expressions of Bim_EL_ in both WCs and SCs of ESCC cell lines, KYSE180 and KYSE410. However, when compared with WCs, the expression of BIM_S_ was markedly increased in SCs treated with 5 mM of ASA (Fig. 5b). Since Bim_S_ represented the most cytotoxic isoforms of Bim, these results suggested that ASA treatment resulted in more efficient cell killing in SCs. Consistently, genome browser view of ATAC-seq revealed that ASA treatment caused a peak to disappear in Bim enhancer region in SCs, indicating that alteration of chromatin structure and accessibility of *Bim* gene by ASA played a role in regulating Bim expression (Fig. 5c).

To determine whether Bim played a key role in controlling cell death and enhancing DDP-induced apoptosis, we employed small-interfering RNA targeting Bim in KYSE180 and KYSE410 cells and examined the impact on synergistic inhibition by ASA and DDP (Fig. 5d). Knockdown of Bim attenuated the chemotherapy sensitization effect of ASA in DDP-treated cells (Fig. 5e, f). Consistently, cell viability assay showed that the inhibition of ASA was significantly attenuated by Bim knockdown in KYSE180-SCs (Supplementary Fig. S5A, B). Moreover, Bim knockdown significantly decreased the expression of cleaved caspase-3, which was up-regulated by ASA (Supplementary Fig. S5C).

Immunohistochemistry assays demonstrated that the xenograft tumours generated by KYSE410 in nude mice highly expressed Bim upon ASA or DDP/ASA treatment compared to controls (Fig. 5g). Taken together, these results indicated that the induction of cell death by ASA or DDP/ASA in ESCC cells, especially SCs, was dependent, at least in part, on the upregulation of proapoptotic Bim gene expression (Supplementary Fig. S5D).

### ASA prevented the occurrence of ESCC in an NMBzA-induced rat ESCC model

CSCs play an important role in tumour initiation [44]. Since the inhibitory effect of ASA on pCSCs, we examined whether ASA could be a potential chemopreventive drug for ESCC. Previously, we developed an NMBzA-induced ESCC rat model and demonstrated that it was a valuable animal model for simulating ESCC progression and chemoprevention [24]. Hence, we determined the effects of ASA in the development of ESCC in the NMBzA-induced rat ESCC model. F344 rats were treated with DMSO (NC) and NMBzA (0.30 mg/kg subcutaneously (s.c.), positive control) three times per week for 25 or 35 weeks to induce the development of ESCC. The NMBzA plus ASA group was offered ASA (2 g/L) in their drinking water as the NMBzA injection started (for details, see ‘Methods’, and Supplementary Fig. S6A). The three groups of rats showed a steady increase in body weight and there were no significant differences up to week 30 (Supplementary Fig. S6B, *P* > 0.05). However, after week 30, rats of the NMBzA-treated group started to lose weight due to obstructive tumour formation in oesophagi, whereas rats in the NMBzA plus ASA-treated group and NC group maintained their weight (Supplementary Fig. S6B). The mortality rate of the NMBzA-treated group was 29.2% (7/24), while the mortality rate of the NMBzA plus ASA-treated group was 0% (0/24) at week 35 (Supplementary Fig. S6C, *P* < 0.05). The rats in the negative group also remained healthy at week 35. These results indicated that the body weight and vital signs of rats were not affected by ASA treatment.

**Fig. 6:**
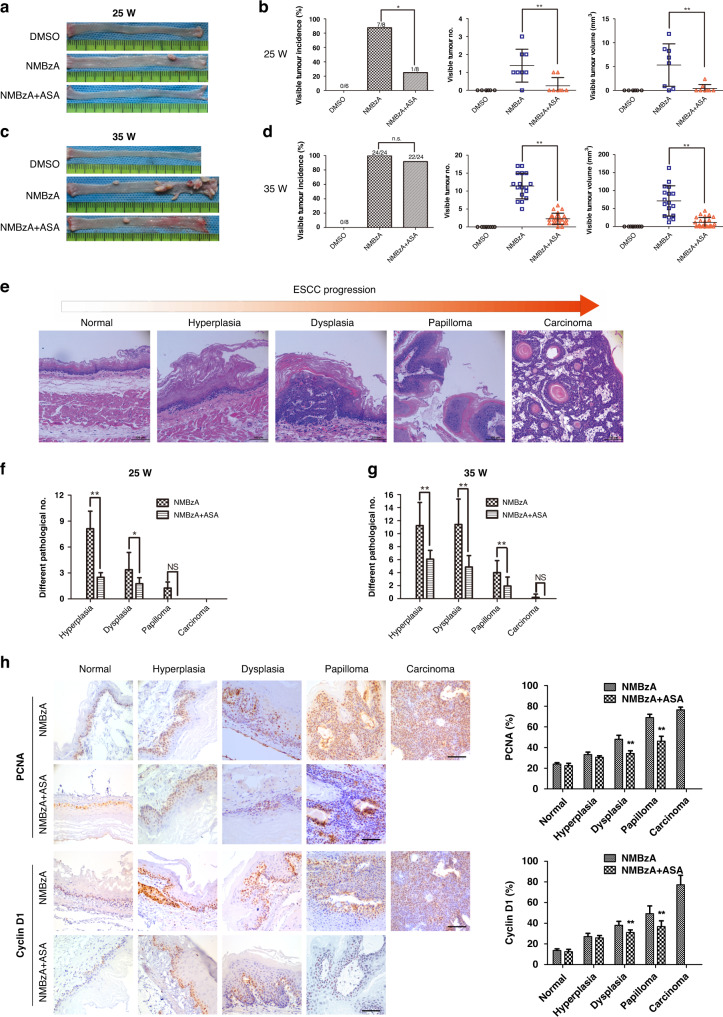
ASA prevents carcinogenesis of ESCC. **a, b** Images of the oesophageal tissues of rats from DMSO, NMBzA or NMBzA+ASA group at week 25 (**a**) and tumour burden of each group (**b**). **c**, **d** Images of the oesophageal tissues of rats from DMSO, NMBzA or NMBzA+ASA group at week 35 (**c**) and tumour burden of each group (**d**). **e** Pathological progression from normal oesophageal epithelium to ESCC by HE staining. **f**, **g** ASA prevents lesions of all stages caused by NMBzA at week 25 (**f**) and week 35 (**g**). **h** IHC staining of PCNA and cyclin D1 in different pathological lesions of rat oesophagus in each group. Positive rates were calculated from six non-contiguous, randomly selected fields of each section. Scale bars, 100 μm. In all the panels, error bars indicate means ± SD; *p < 0.05, **p < 0.01; n.s., not significant. Student’s *t* test.

Oesophageal tumours were visualized in rats receiving NMBzA at weeks 25 and 35 (Fig. 6a, c). At week 25, tumours were observed in 87.5% of the animals from the NMBzA group. In comparison, tumour incidence was significantly lower in the NMBzA plus ASA group (12.5%). Tumour multiplicity and tumour volumes were also significantly decreased by ASA treatment at week 25 (Fig. 6b). At week 35, there was no significant difference in tumour incidence between the two groups (NMBzA group: 100%; NMBzA plus ASA group: 91.7%). However, the counts and volumes of tumours were significantly reduced by ASA (Fig. 6d). The pathological progression of ESCC in the rat model generally evolved from normal epithelium to hyperplasia, dysplasia, papilloma and carcinoma (Fig. 6e). Histopathological diagnosis revealed that, at week 25, the quantities of hyperplasia and dysplasia were significantly decreased in the NMBzA plus ASA-treated group in comparison to the NMBzA-treated group (Fig. 6f). At week 35, decreased quantities of hyperplasia, dysplasia and papilloma in the oesophagus of the NMBzA plus ASA-treated group were observed (Fig. 6g). Consistently, immunohistochemistry analyses indicated that the preventive effects of ASA resulted in the decreased expression of cell proliferation markers cyclin D1 and PCNA (Fig. 6h). The expression of Bim was also examined by immunohistochemistry, but no significant increase was observed in the NMBzA plus ASA-treated group. We speculated that, under the ASA treatment, the pCSCs with high expression of Bim already had apoptosis. As a result, it could not progress to the carcinoma stage. Notably, ASA could reduce protein expression levels of COX-2, suggesting that the anti-inflammatory function of ASA may also play a part in anti-tumour effect (Supplementary Fig. S6D). Taken together, these results demonstrated that ASA treatment delayed the development of precancerous lesions and ESCCs in the NMBzA-induced rat ESCC model.

## Discussion

Despite the revelation of the genomic landscape and recent improvements of therapy, the progress in treatment and improving prognosis of ESCC was still lagging behind many other cancers. Lack of targeted drugs, the resistance of conventional therapy and recurrence of tumour were the main causes of poor outcomes of ESCC. DDP was the most widely used chemotherapeutic drug against ESCC; its objective response rate (ORR) was only ~30% [45, 46]. Previously, many studies showed that combining ASA with chemotherapeutic drugs significantly enhanced cytotoxic response and suppressed cancer growth [47–49]. Likewise, in this study, we found that ASA dramatically enhanced the cytotoxicity of DDP in human ESCC cell lines in vitro and in vivo. The strong inhibitory effects of ASA were on stemness properties of ESCC cells, indicating that ASA impacted upon CSCs of ESCC. These effects of ASA may be a consequence of induction of CSC differentiation or directly inducing cell death in CSCs. Further apoptotic experiments proved that ASA could enhance the sensitivity of ESCC cells to DDP by inducing the death of pCSC cells. Our results showed that ASA inhibited cell proliferation, suppressed sphere formation, perturbed stemness-associated genes expression and affected chromatin structure of ESCC. By chromatin remodelling, ASA altered the expression of massive genes, especially pro-apoptosis gene Bim, resulting in significant cell death. Moreover, animal model experiments demonstrated that ASA reduced the incidence of NMBzA-induced rat ESCC and precancerous lesions. These results, together with previous epidemiological and clinical studies [15–17], suggested that the combination of chemotherapy and ASA treatment of ESCC patients might improve their outcomes by effectively targeting CSCs.

A large body of evidence demonstrated that ASA exerted its antitumour capability via the irreversible inhibition of COX-2 activity [14, 50, 51]. However, unlike the treatment of ASA, treatment of NS-398, a selective inhibitor of COX-2, did not promote cell death and inhibit sphere formation significantly, suggesting that the effect of ASA was likely attributed to COX-2-independent pathways in ESCC. Interestingly, we found that ASA could reduce the efflux activity of ESCC cells by inhibiting PI3K signalling, which was reported to mediate P-glycoprotein (P-gp) expression and enhance DDP resistance [52]. This suggested that inhibition of PI3K signalling by ASA may contribute to overcoming DDP resistance.

Given the fact that ASA was a unique non-steroidal anti-inflammatory drug (NSAID) that possessed an acetyl group, ASA could target and acetylate many cellular proteins to exert its anti-tumour functions. Recently, several studies demonstrated that ASA could display epigenetic effects through directly or indirectly acetylating histones, leading to extensive chromatin remodelling and gene transcriptional level alterations [19, 53–55]. Our results also found that ASA could cause extensive acetylation in histone H3 in SCs. Current studies in colorectal cancer showed that the effect of ASA on histone acetylation is mainly through the indirect mechanism, such as p300-mediated enzyme reaction [19] or inhibition of the activity of histone deacetylases (HDACs) [56]. Nevertheless, the mechanisms involved in the control of histone acetylation by ASA in ESCC cells (especially SCs) should be investigated in the future. Consistently, our ATAC-seq experiments demonstrated that treatment of ASA in ESCC cells led to profound alterations in chromatin remodelling and transcription of genes. Importantly, it was more prominent for ASA to target the pCSCs of ESCC cells, resulting in more accessible chromatin regions of pCSCs. Gene enrichment analysis showed that ASA significantly affected proliferation-related pathways (e.g. PI3K/Akt pathway) and other biological regulation processes. As previous studies demonstrated that active epigenetic regulations mediated the maintenance of CSC characteristics [57–59], although the cause of preferential effects of ASA on pCSCs of ESCC cells remained to be further explored, we speculated that it could be related to the original epigenetic state of pCSCs.

Our ATAC-seq study identified that the chromatin regions of *Bim* gene were remodelled upon ASA treatment in ESCC cells, especially in pCSCs, resulting in increases in Bim expression. As a proapoptotic protein, Bim functioned as a key molecule by binding and antagonising anti-apoptotic members of Bcl-2 family (such as Bcl-2) [60]. Hence, increases in Bim expression could mediate and further promote the death of pCSCs treated with ASA and ASA/DDP in ESCC. Previous studies showed that low expression of Bim was closely related to the resistance of epidermal growth factor receptor tyrosine kinase inhibitor (EGFR-TKI) in non-small cell lung carcinomas (NSCLCs) [61]. Vorinostat, an HDAC inhibitor (HDACi), epigenetically activated the expression of Bim and restored the sensitivity of NSCLC cells to EGFR-TKI [62]. Since both ASA and vorinostat were able to induce histone hyperacetylation, ASA might promote transcriptional activation of Bim through the similar epigenetic regulation of vorinostat. Thus, we speculated that ASA regulated epigenetic alterations and chromatin remodelling by promoting the acetylation of histones/nucleosomes and caused the transcriptional activation/inactivation of critical cellular genes, especially the proapoptotic gene Bim, ultimately leading to the death of pCSCs in ESCC.

Consistent with the results obtained from human cell lines in vitro and in vivo, preventions of ESCC development and progression by ASA were also demonstrated in an NMBzA-induced ESCC rat model. We showed that ASA significantly reduced the incidence of NMBzA-induced rat ESCC and precancerous lesions in high-dose (0.3 mg/kg for 25 or 35 weeks) NMBzA-treated rats (see ‘Methods’). In contrast, previous studies showed that L-748706, a selective cyclooxygenase-2 inhibitor, displayed protective effects of ESCC development and progression in low-dose (0.25 mg/kg for 5 weeks) NMBzA-treated rats, but insufficiently protected ESCC development and progression in high-dose (0.5 mg/kg for 5 weeks) NMBzA-treated rats [63]. This suggested that inhibition of COX-2 might be only partially involved in the prevention of NMBzA-induced ESCC. Hence, our animal model experiments further revealed that ASA would have more wide-ranging effects other than the specific inhibition of COX-2 activity on chemoprevention in ESCC development and progression. In addition, this model could be further applied to investigate the sensitizing effect of ASA on chemotherapy (e.g. combined treatment with DDP).

In summary, our study demonstrated the inhibition of pCSCs by ASA in ESCC, which led to the abrogation of the chemoresistance and carcinogenesis of ESCC. Given the lack of effective and specific drugs targeting CSCs of ESCC at present, ASA, a most common and widely used drug, would be a promising prospect for clinical translation in the treatment and prevention of ESCC.

## Supplementary information

Checklist

Supplementary material

ARRIVE checklist

Supplementary table S2

Supplementary table S3

## Data Availability

All data presented within the article and its Supplementary information files are available upon request from the corresponding author. The ATAC-seq raw data files have been deposited on GSA for humans (HRA001008).
