# Supplementary material for: Aspirin enhances the therapeutic efficacy of cisplatin in oesophageal squamous cell carcinoma by inhibition of putative cancer stem cells
Source: Br J Cancer. 2021 Jul 27;125(6):826–38. doi: 10.1038/s41416-021-01499-3 (PMC8438052; doi:10.1038/s41416-021-01499-3)
Supplement: Supplementary file 2 — Supplementary material [file 41416_2021_1499_MOESM2_ESM.pdf]

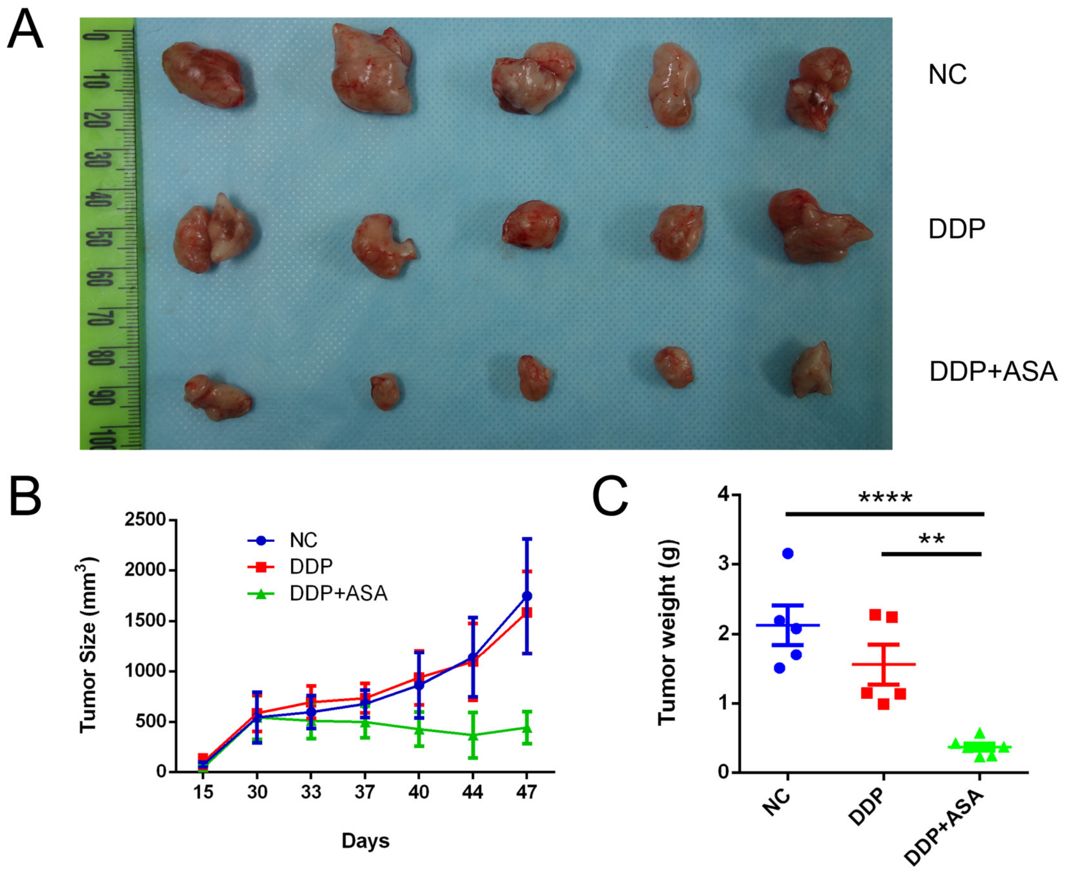

Fig. S1 ASA promotes the DDP treatment in KYSE150 xenograft model. (A) Images of KYSE150 xenografts subjected to the indicated treatments: ASA (2 g/L in drink water), DDP (5 mg/kg) or the combination during 30 days. **(B-C)** Tumor volume (B) and weights (C) quantification of KYSE150 xenografts (error bars indicate SD, n=6, **P<0.01, ***P<0.001, Student t test).

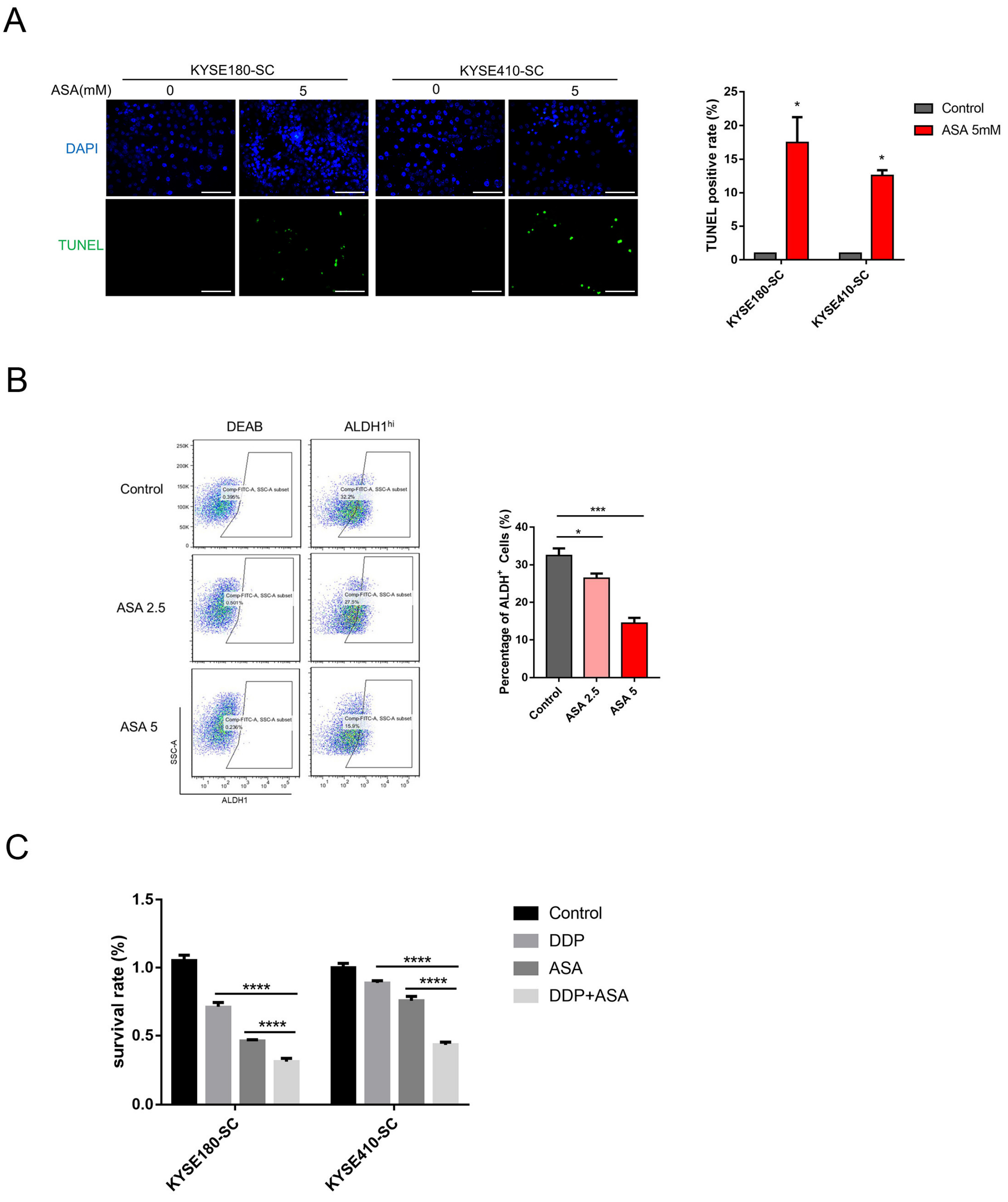

Fig. S2 ASA induces cell death in pCSCs of ESCC. (A) Representative images of TUNEL assay (left) and quantification (right) show the percentage of apoptotic cells of SCs following treatment with 5mM ASA for 48h (n=3). Scale bar, 50 μm. **(B)** Percentage of $ALDH^{hi}$ cells of KYSE180 cell line treated with ASA for 24h was detected by ALDEFLUOR assay. DEAB (ALDH inhibitor) was used to provide a negative control (n=3). **(C)** The survival rate of SCs of ESCC cells treated with 5μg/ml DDP, 5mM ASA or the combination for 24h (n=3). In all the panels, error bars indicate means ± SD; $*p < 0.05$, $***p < 0.001$, $****p < 0.0001$. Student's t test.

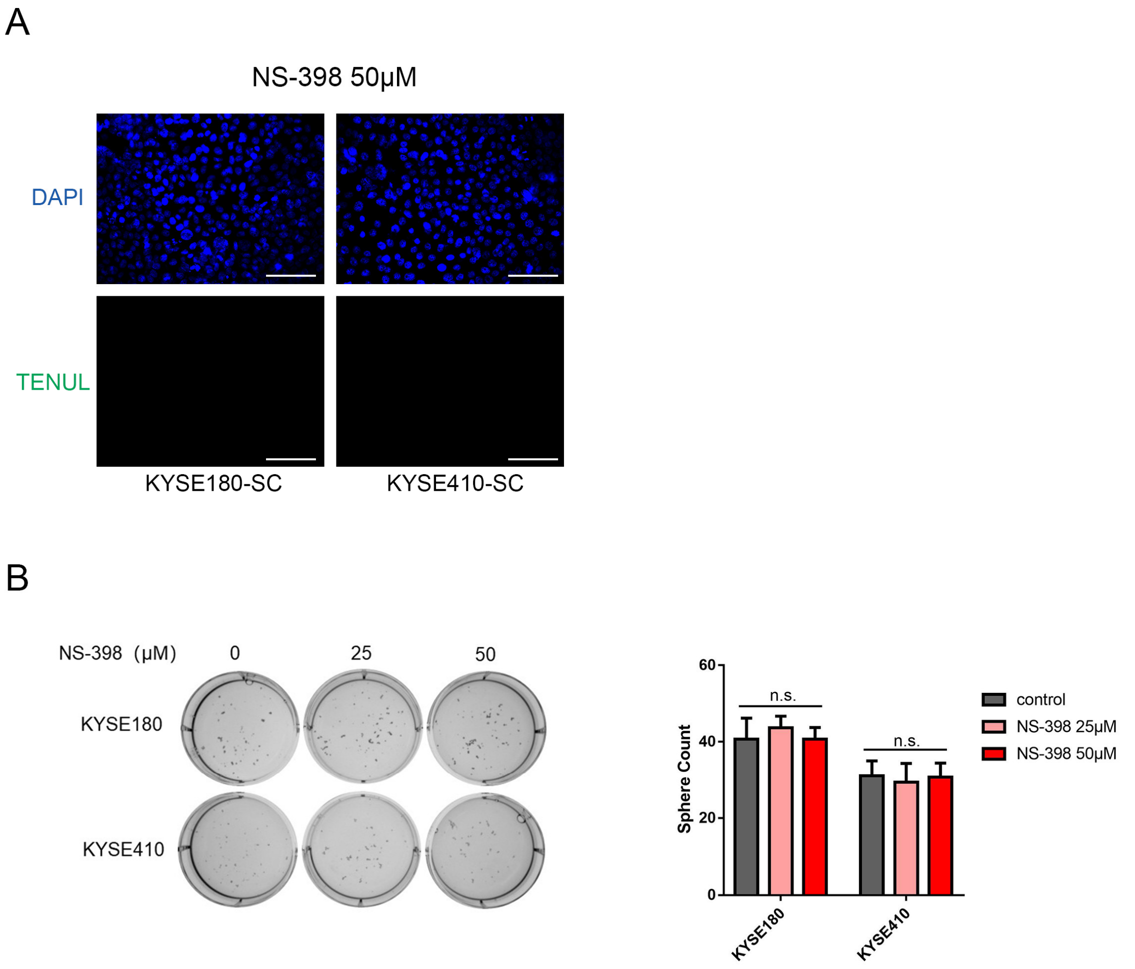

Fig. S3 COX-2 inhibitor cannot reduce pCSCs properties and promote cell death in ESCC. (A) Representative images of TUNEL assay of SCs following treatment with 50μM NS-398 for 48h. Scale bar, 50 μm. **(B)** Representative image (left) and quantification (right) of spheres formed by KYSE180 and KYSE410 cells exposed to indicated concentrations of NS-398.

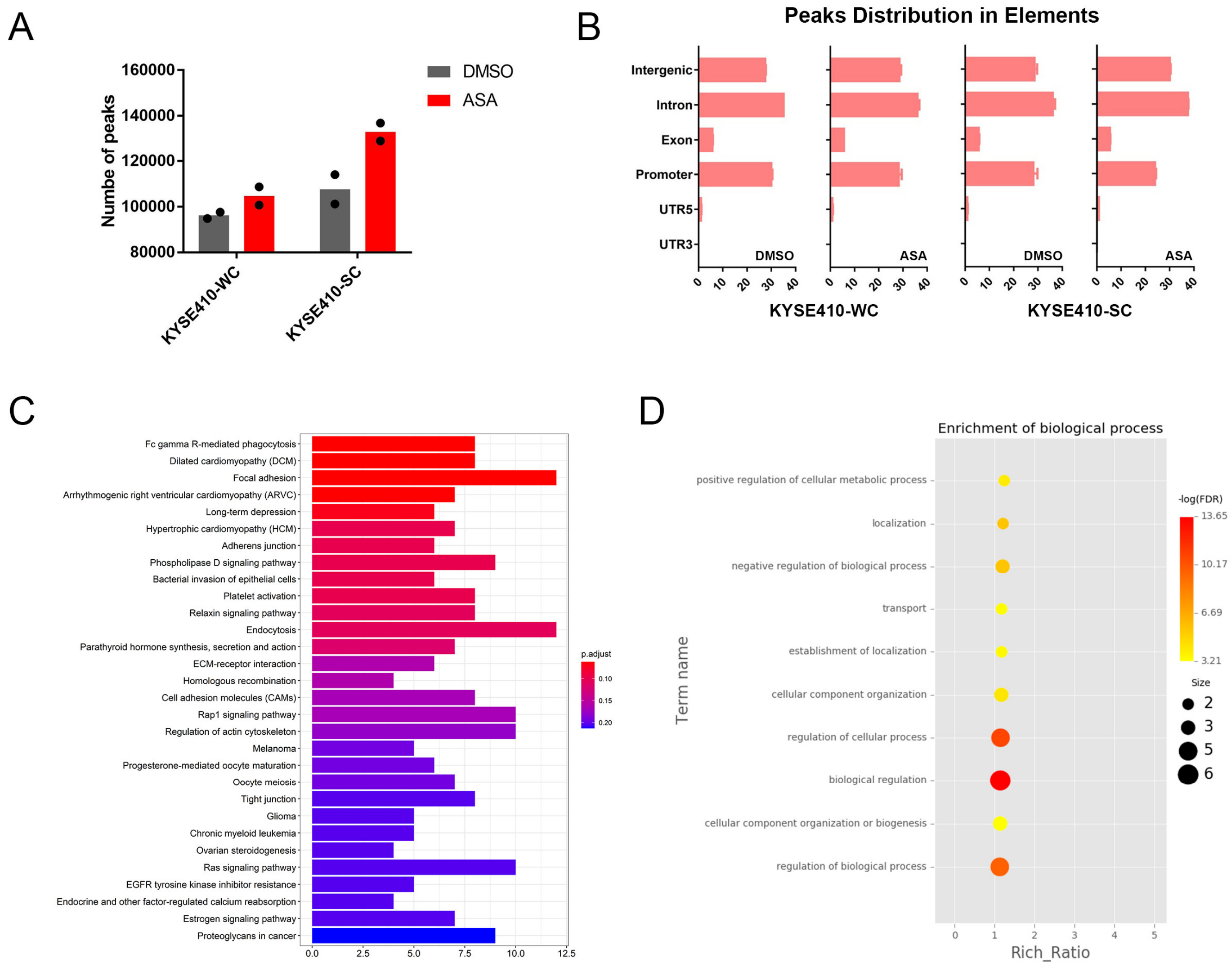

Fig. S4 Chromatin accessibility is altered by ASA in KYSE410 cell line. (A) Number of ATAC-seq peaks detected in WCs and SCs following treat with DMSO or ASA (detected in 2 replicates). **(B)** Distribution of ATAC-seq peaks in WCs and SCs to genomic regions. **(C)** KEGG pathway analysis among genes altered by ASA for chromatin accessibility in WCs. **(D)** Gene ontology analysis among genes altered by ASA for chromatin accessibility in SCs. The size of each dot indicates the number of genes enriched into the GO term and "Rich Ratio" means the number of genes annotated to the GO term accounts for the proportion of all genes.

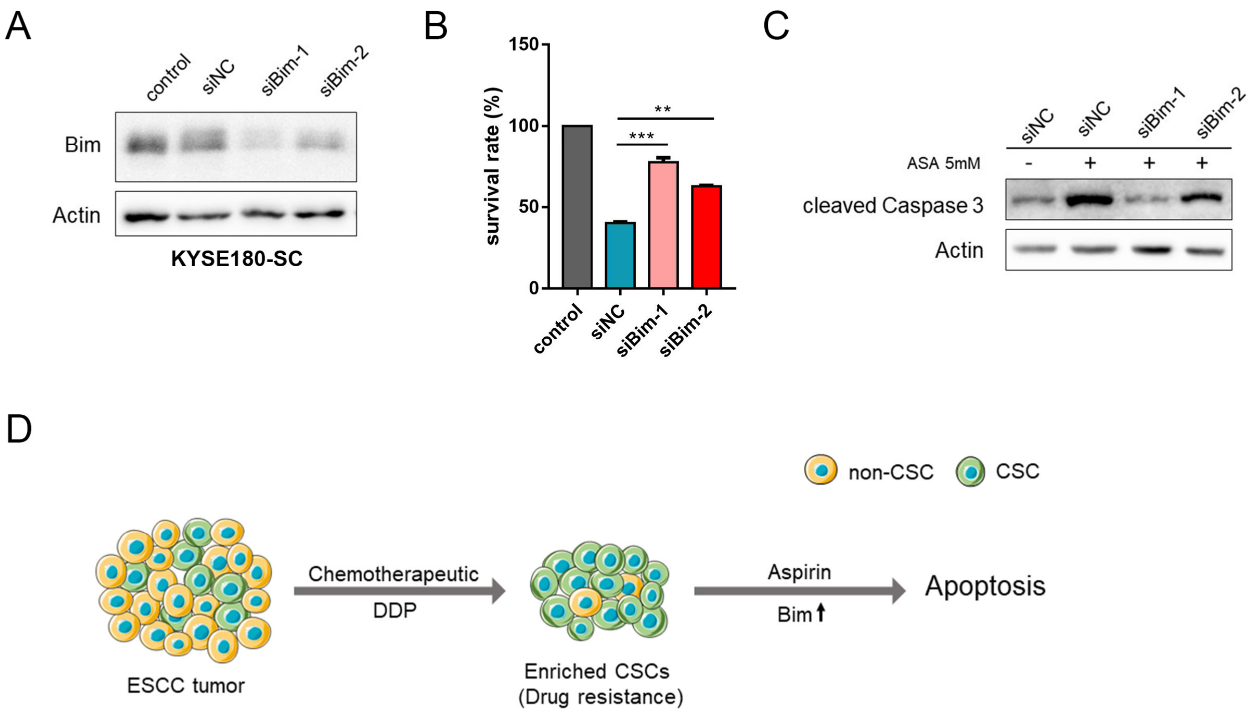

Fig. S5 Knockdown of Bim attenuates the pro-apoptotic effect of ASA in KYSE180-SC cells. (A) Western blot analysis confirming siRNA-mediated Bim knockdown in KYSE180-SC cells. **(B)** The survival rate of KYSE180-SCs treated with 5mM ASA for 48h after Bim knockdown (error bars indicate SD, n=3, **P<0.01, ***P<0.001, Student's t test). **(C)** Immunoblotting for cleaved caspase3 in KYSE180-SCs treated with 5mM ASA for 48h after Bim knockdown. **(D)** Pattern diagram of ASA in promoting the therapeutic effect of DDP in ESCC.

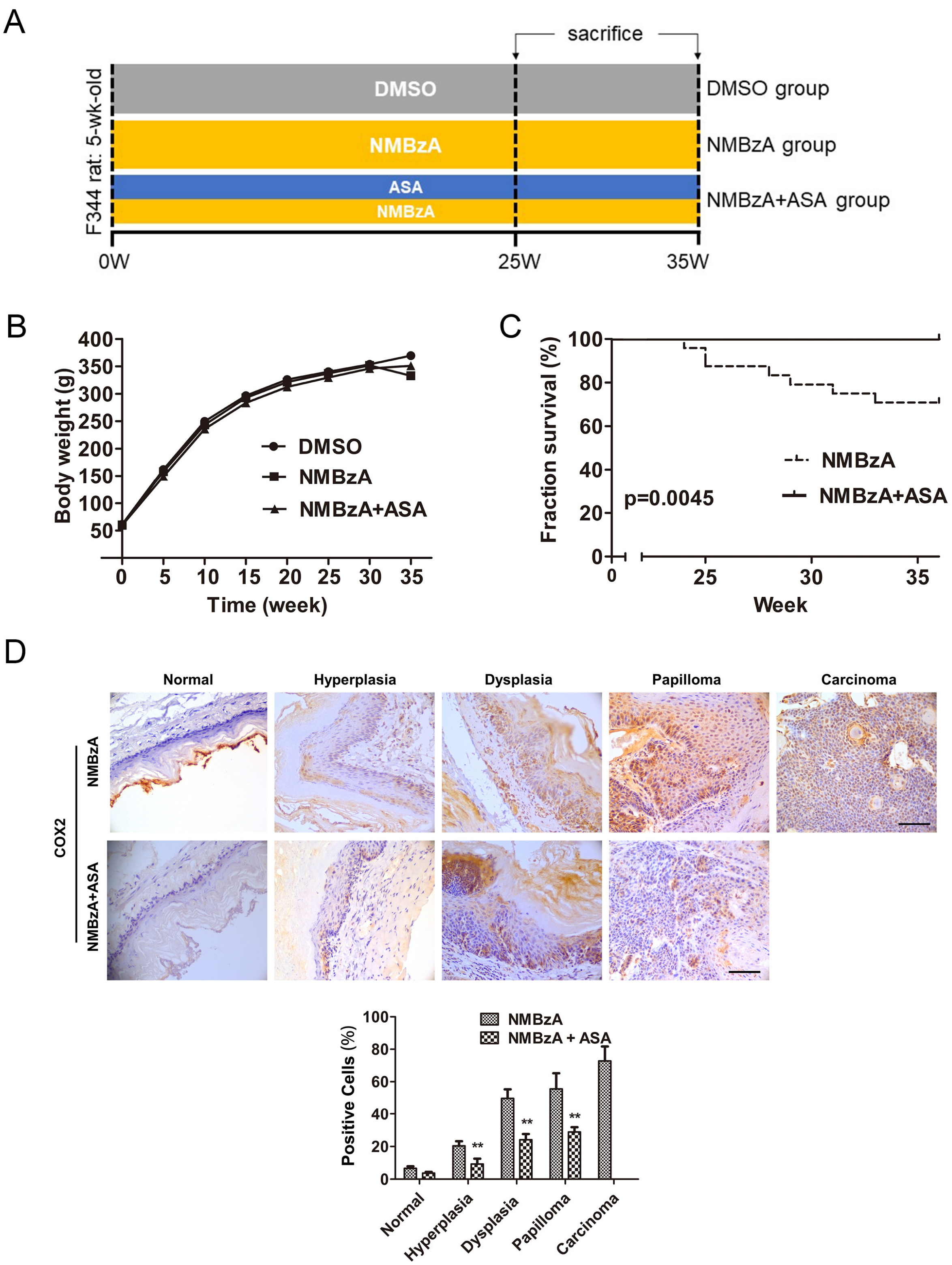

Fig. S6 ASA suppresses esophageal carcinogenesis in rat ESCC model. (A) Study design of rat ESCC model. **(B)** Body weights of rats are measured during the growth in each group. **(C)** The survival fraction of rat in NMBzA group and NMBzA + ASA group. **(D)** IHC staining of COX-2 in different pathological lesions of rat esophagus in each group. Positive rates were calculated from 6 non-contiguous, randomly selected fields of each section. Scale bars, 100 μm. Error bars indicate means ± SD; $^{**}p < 0.01$. Student's t test.

Supplement Table S1. The Q-PCR primers used in this study.

Primer	Sequence (5'to3')
Sox2_F	ACACCAATCCCATCCACACT
Sox2_R	GCAAACTTCCTGCAAAGCTC
OCT4_F	GGACCAGTGTCCTTTCCTCT
OCT4_R	CCAGGTTTTCTTTCCCTAGC
Nanog_F	TGAACCTCAGCTACAAACAG
Nanog_R	TGGTGGTAGGAAGAGTAAAG
Bmi-1_F	TCGTTCTTGTTATTACGCTGTTT
Bmi-1_R	CGGTAGTACCCGCTTTTAGGC
ABCA3_F	AGAAATACGGTGCCGGCTATCACA
ABCA3_R	CAATGCCCAGCTCTTTCTGCTTCT
ABCB1_F	GCTCCTGACTATGCCAAAGC
ABCB1_R	TCTTCACCTCCAGGCTCAGT
ABCC1_F	CTGGGCTTATTTCGGATCAA
ABCC1_R	TGAATGGGTCCAGGTTCATT
ABCG2_F	CACCTTATTGGCCTCAGGAA
ABCG2_R	CCTGCTTGGAAGGCTCTATG
BCL2L1_F	GAGCTGGTGGTTGACTTTCTC
BCL2L1_R	TCCATCTCCGATTCAGTCCCT
BCL2L11_F	GCTACCAGATCCCCGCTTTT
BCL2L11_R	CCTGCCTCATGGAAGCCATT
BCL2A1_F	TTGTGTCCGTAGACACTGCC
BCL2A1_R	GGGGCAATTTGCTGTCGTAG
TRAF1_F	TGAGAGGGGAGTATGATGCG
TRAF1_R	GACGCTGAGCTTAGGTCAGG
GAPDH_F	CACCTTATTGGCCTCAGGAA
GAPDH_R	CTCCACGACGTACTCAGCG

Supplement Table S2. MAnorm analysis results of ATAC-Sequencing of KYSE410-SC and KYSE410-WC following ASA treatment. Unique_peak1 represent peaks opened in ASA-treatment, unique_peak2 represent peaks closed in ASA-treatment.

Supplement Table S3. Annotation of specific peak related genes with changed chromatin accessibility upon ASA treatment in KYSE410-SC and KYSE410-WC. Unique_peak1 represent peaks opened in ASA-treatment, unique_peak2 represent peaks closed in ASA-treatment.
